# Supplementary material for: A fluorogenic cyclic peptide for imaging and quantification of drug-induced apoptosis
Source: Nat Commun. 2020 Aug 12;11:4027. doi: 10.1038/s41467-020-17772-7 (PMC7423924; doi:10.1038/s41467-020-17772-7)
Supplement: Supplementary file 1 — Supplementary Information [file 41467_2020_17772_MOESM1_ESM.pdf]

Electronic Supplementary Information

A fluorogenic cyclic peptide for imaging and quantification of drug-induced apoptosis

Table of Contents

Supplementary Figures

Supplementary Tables

Supplementary Discussion

Supplementary Movies

Supplementary Methods

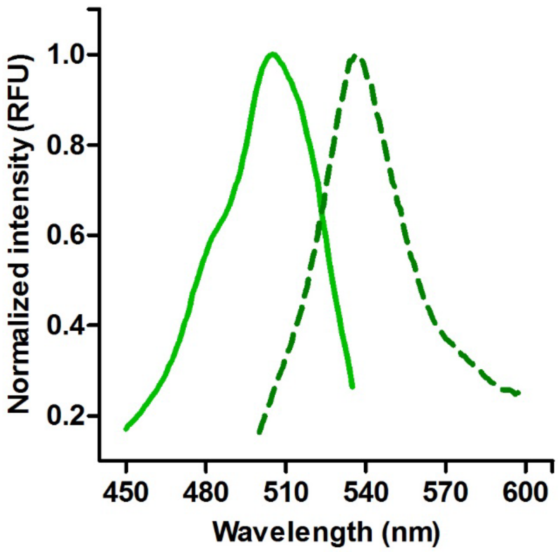

Supplementary Figure 1. Normalized absorbance (solid line) and fluorescence emission (dashed line) spectra of **Apo-15** (50 μM) in PBS (pH 7.4) (n=3 independent measurements). Source data are provided as a Source Data file.

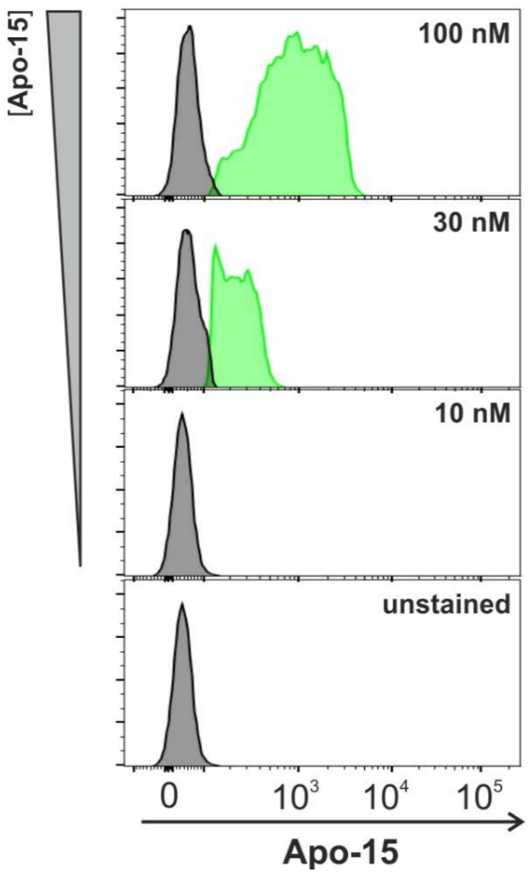

Supplementary Figure 2. Dose-dependent binding of **Apo-15** to *in vitro* cultured human neutrophils (to induce apoptosis) following incubation with the indicated concentrations for 10 min at r.t. ($\lambda_{exc.}$: 488 nm, $\lambda_{em.}$: 525 nm). Representative histograms show **Apo-15** binding to viable (gray - Annexin V negative) cells and apoptotic (green - Annexin V positive) cells (n=5).

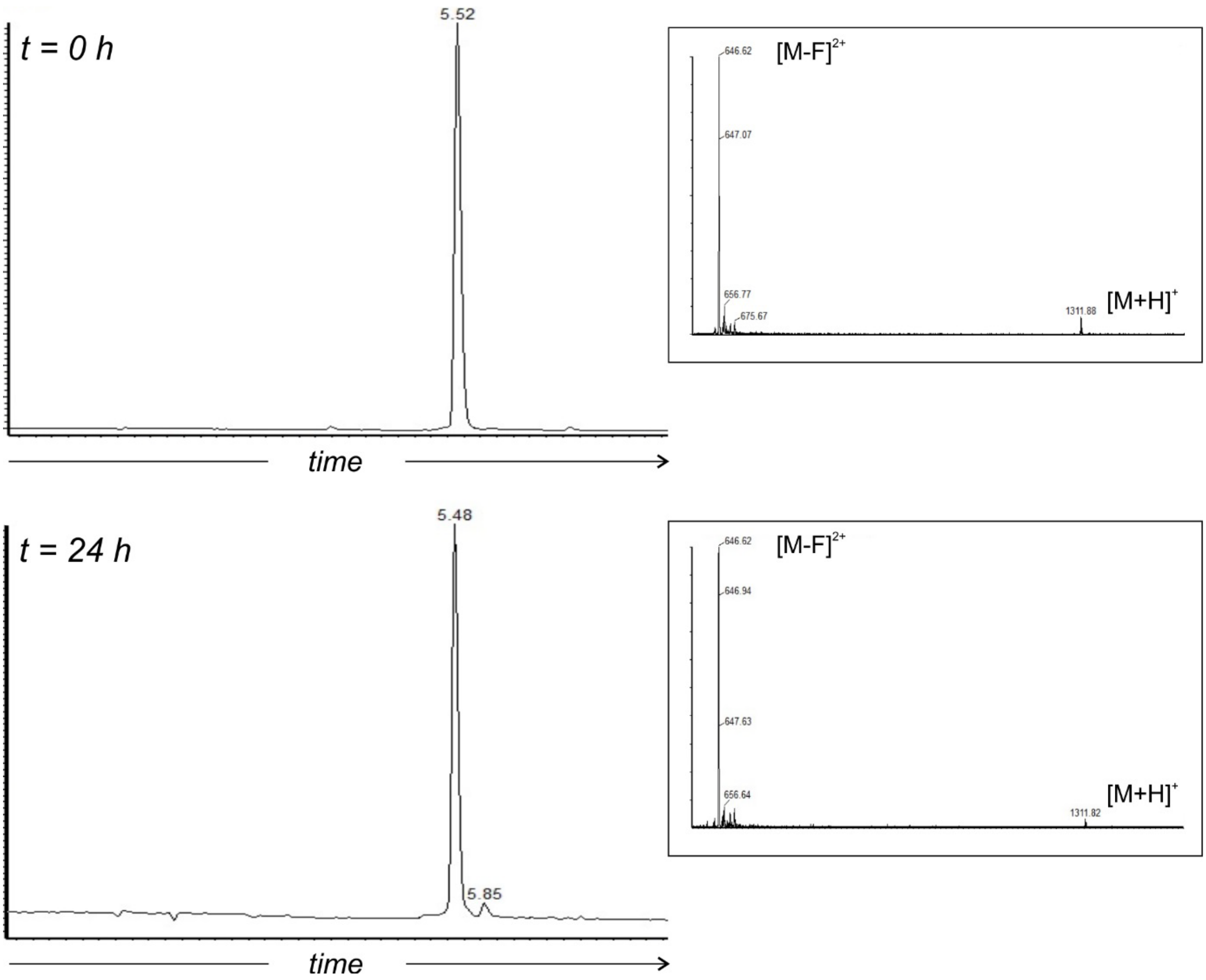

Supplementary Figure 3. HPLC traces of **Apo-15** (50 μM) before (upper panel) and after treatment (lower panel) with a protease cocktail isolated from *Streptomyces griseus* at 37 ºC (5 μg mL^{-1}, pH = 7.1). Insets show mass spectra (ESI+) of the main HPLC peak under normal and proteolytic conditions. The chemical purity of **Apo-15** at 0 h and 24 h was determined as above 95%.

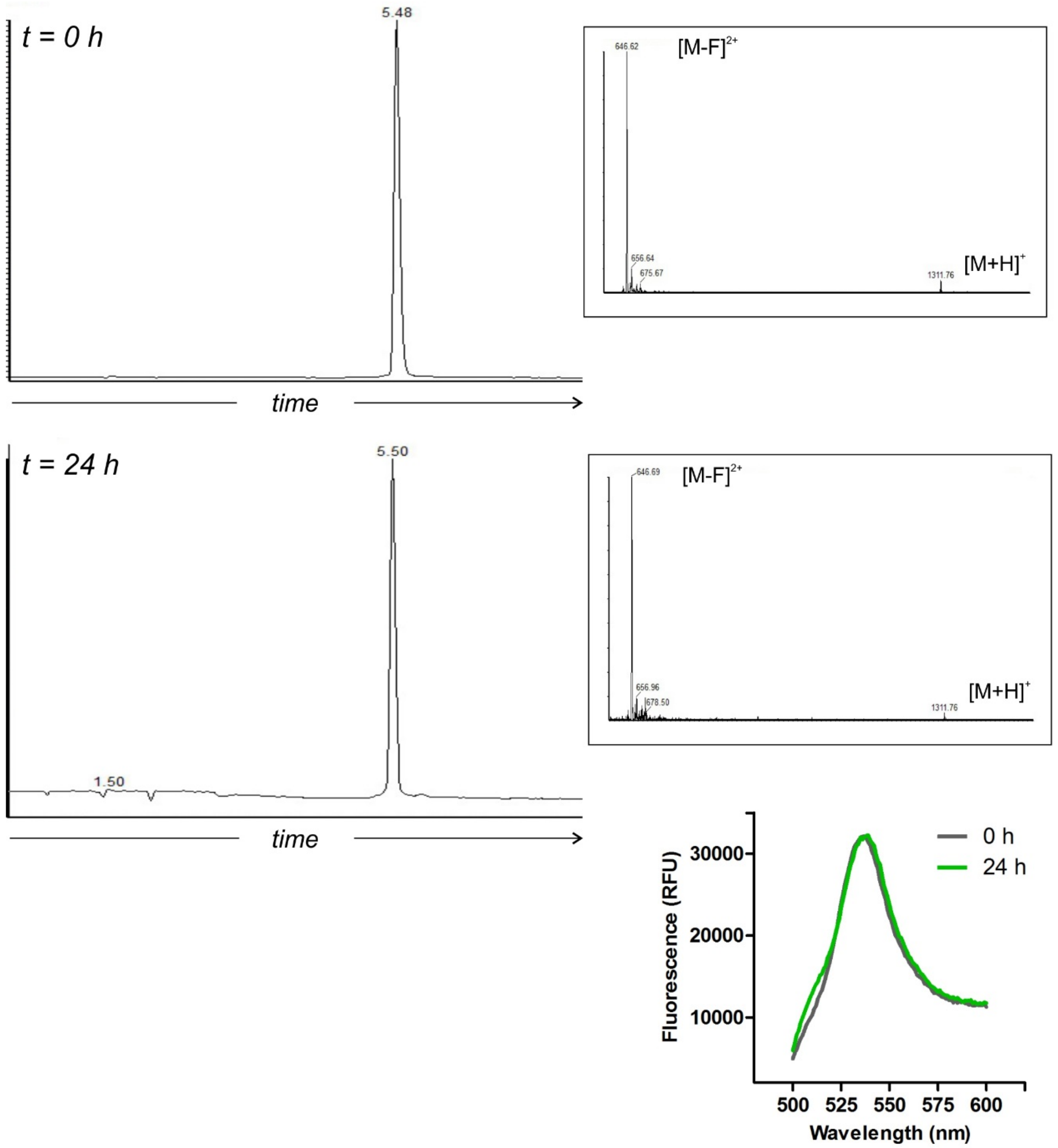

Supplementary Figure 4. HPLC traces of **Apo-15** (50 μM) before (upper panel) and after treatment (lower panel) with H_2O_2 (100 μM, pH=7.1). Insets show mass spectra (ESI+) of the main HPLC peak under normal and oxidative (100 μM H_2O_2) conditions. The chemical purity of **Apo-15** at 0 h and 24 h was determined as above 95%. Emission spectra of **Apo-15** ($\lambda_{exc.}$: 450 nm) before and after H_2O_2 treatment (n=3 independent measurements). Source data are provided as a Source Data file.

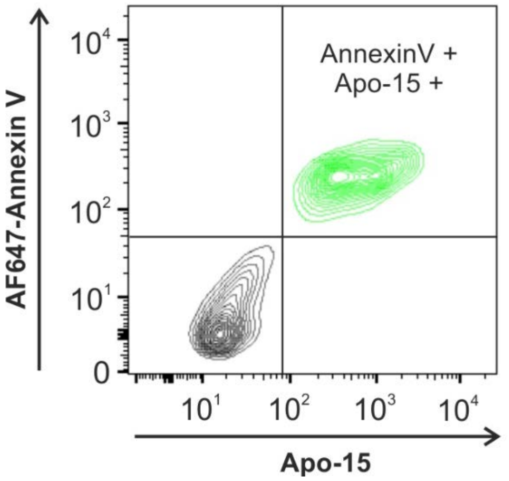

Supplementary Figure 5. Histograms showing binding of **Apo-15** to apoptotic mouse bone marrow-derived neutrophils (BMDN). BMDN were constitutively aged for 18 h to induce apoptosis, followed by incubation with **Apo-15** (100 nM) and AF647-Annexin V (25 nM) for 10 min at r.t. Samples were analysed on a 5L LSR flow cytometer (n= 3 independent experiments).

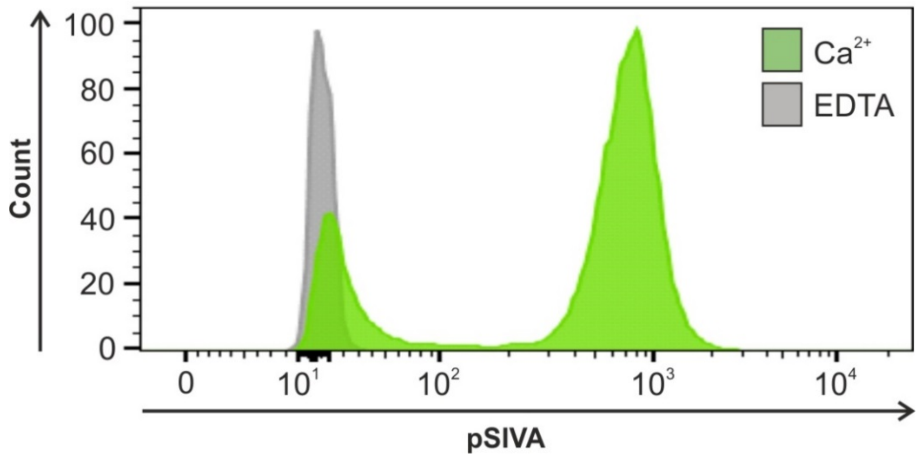

Supplementary Figure 6. Histograms showing pSIVA binding to mouse thymocytes in which apoptosis had been induced by incubation with 800 nM dexamethasone for 6 h. Cells were then washed and re-suspended in 20 mM HEPES/140 mM NaCl buffer containing either $CaCl_2$ (2 mM) (green) or EDTA (2.5 mM) (gray) ($\lambda_{exc.}$: 488 nm, $\lambda_{em.}$: 525 nm) (n=4).

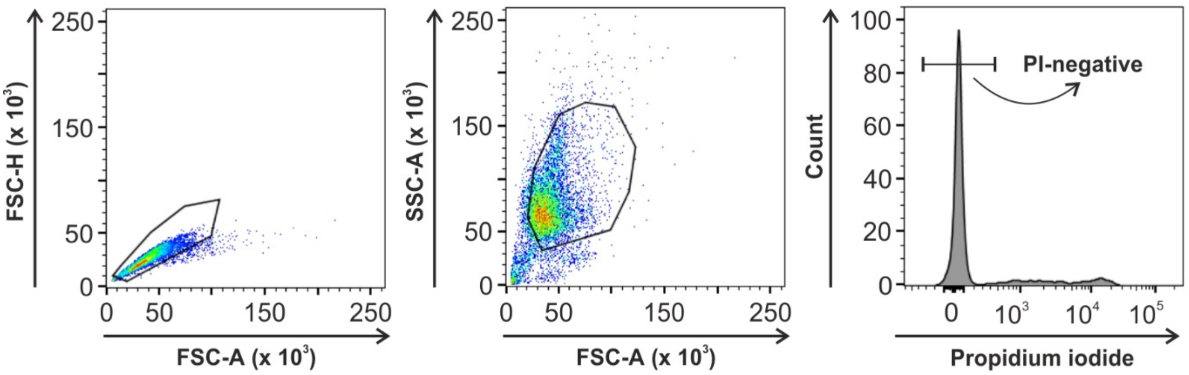

Supplementary Figure 7. Gating strategy prior to cell sorting of **Apo-15**-positive and **Apo-15**-negative cells. Left: single-cell gating; centre: neutrophil gating on FSC-A/SSC-A, right: propidium iodide (PI)-negative cells.

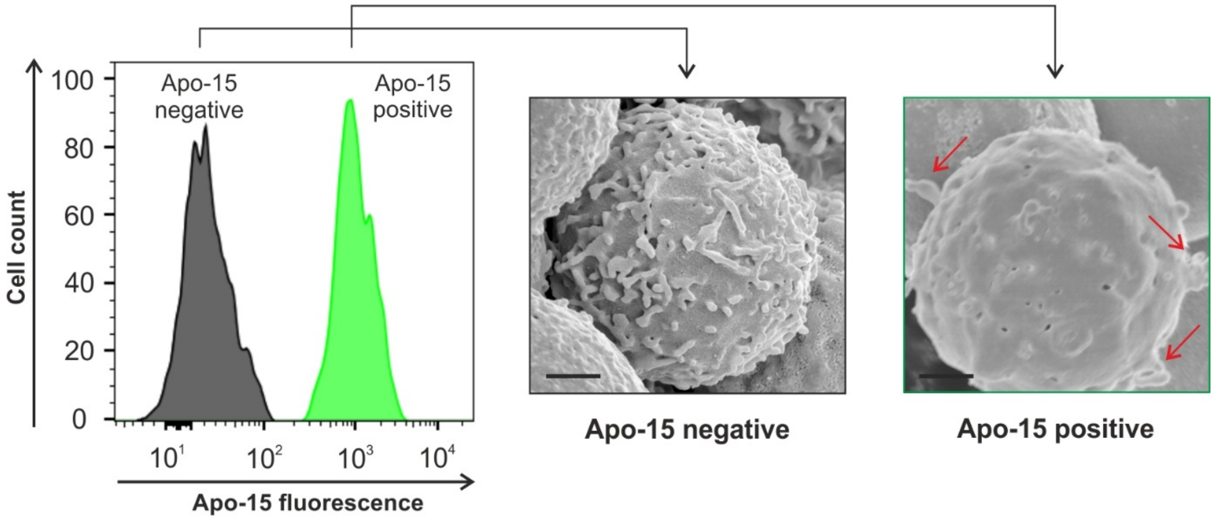

Supplementary Figure 8. Fluorescence-activated cell sorting human neutrophil populations that have undergone apoptosis following in vitro culture for 18 h and incubation with **Apo-15** (100 nM). Sorted **Apo-15**-negative and **Apo-15**-positive populations are represented in the histogram as gray and green profiles, respectively ($\lambda_{exc.}$: 488 nm, $\lambda_{em.}$: 525 nm). Representative morphological analysis (4 independent images from 2 independent experiments) of **Apo-15**-negative (left) and **Apo-15**-positive (right) by scanning electron microscopy. Red arrows highlight blebs in the plasma membrane, which are a characteristic feature of apoptotic cells. Scale bar: 1 µm.

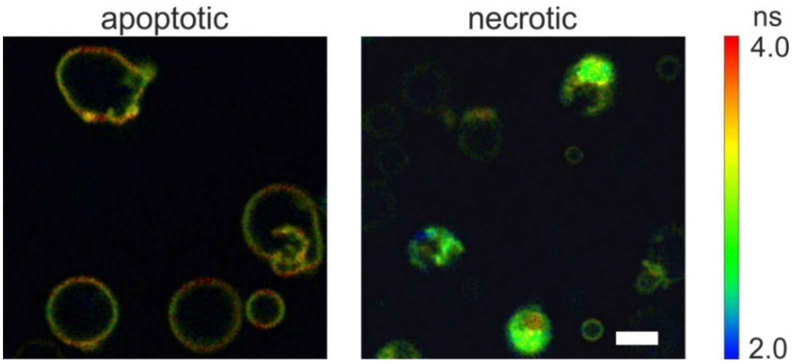

Supplementary Figure 9. Fluorescence lifetime pseudo-color images (from 4 independent images) of **Apo-15-**labeled BL-2 cells at different stages of cell death by UV irradiation (apoptotic: 300 mJ cm^{-2}, necrotic: 900 mJ cm^{-2}). Scale bar: 5 μm.

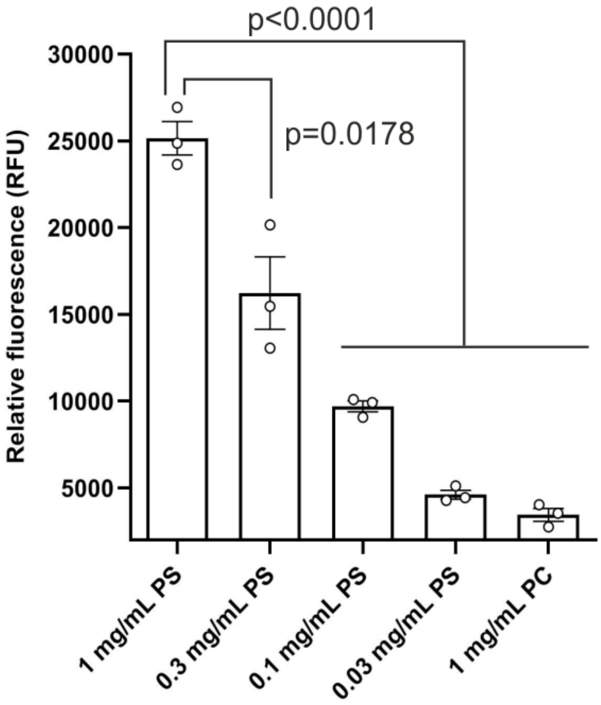

Supplementary Figure 10. Concentration-dependent binding of **Apo-15** (1 μM) to lipid monolayers containing increasing amounts of phosphatidylserine (PS). Phosphatidylcholine (PC) monolayers were used as a negative control. Values represented as mean values±SD (n=3 independent experiments). P values obtained from two-tailed *t* tests. Source data are provided as a Source Data file.

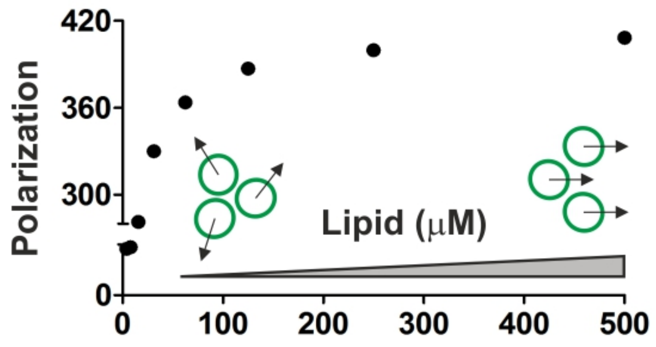

Supplementary Figure 11. Fluorescence polarization plots of PC:PS liposomes with increasing lipid content after labeling with **Apo-15** (500 nM, $\lambda_{exc.}$: 485 nm, $\lambda_{em.}$: 520 nm). Source data are provided as a Source Data file.

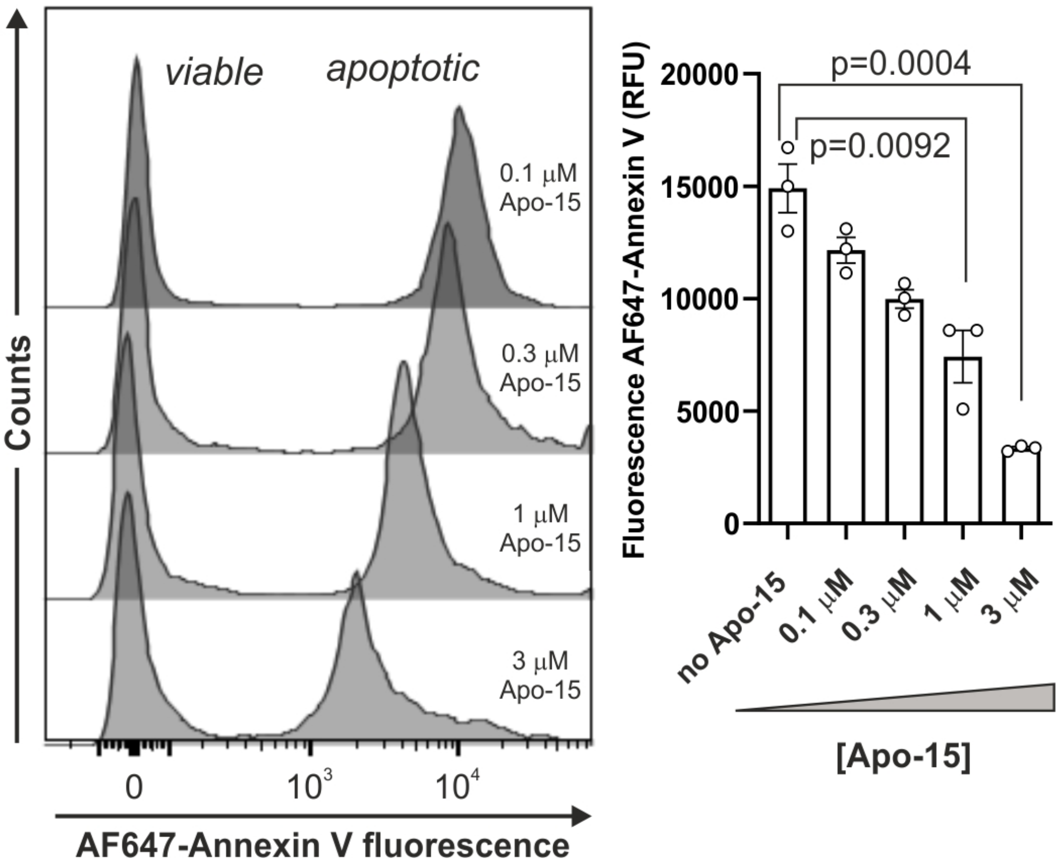

Supplementary Figure 12. Competition between **Apo-15** and Annexin V for binding to apoptotic cells. Histograms show Annexin V (9 nM) binding to viable and apoptotic neutrophils in the presence of increasing concentrations of **Apo-15**. Samples were analysed using a 5L LSR flow cytometer and the mean fluorescence intensity of AF647-Annexin V-labeled apoptotic cells was calculated using FlowJo software and presented as mean values±SEM (n=3 independent experiments). P values obtained from two-tailed *t* tests. Source data are provided as a Source Data file.

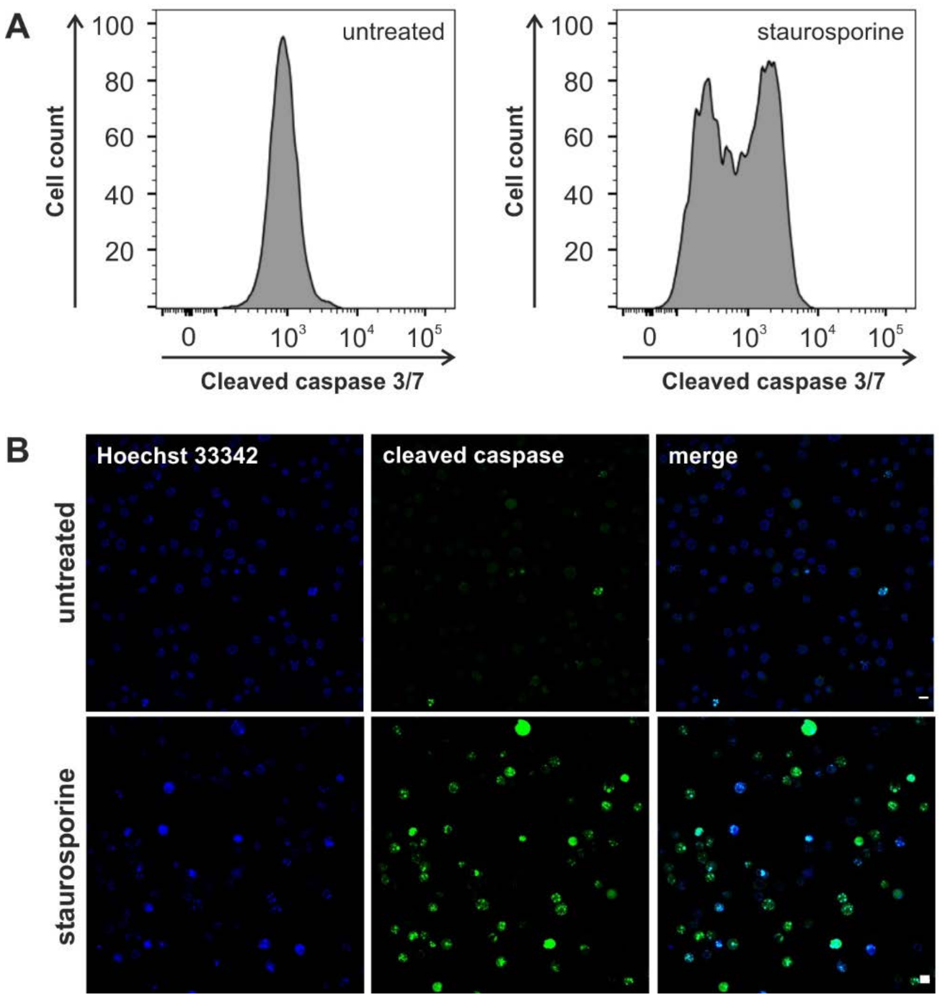

Supplementary Figure 13. A) Representative histograms of PLB-985 cells before and after treatment with staurosporine (1 µM, 6 h) and subsequent staining with CellEvent cleaved caspase-3 (7.5 µM, 30 min) (n=3). B) Fluorescence confocal microscopy images (3 independent images from 2 independent experiments) of PLB-985 cells that had been treated or not with staurosporine (1 µM, 6 h). Cells were stained with Hoechst 33342 (blue) as a nuclei marker and CellEvent to detect cleaved caspase-3 (green). Scale bar: 5 µm.

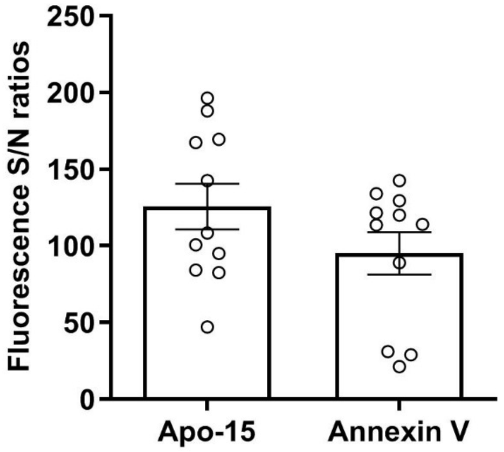

Supplementary Figure 14. Quantification of the fluorescence signal-to-noise (S/N) ratios in apoptotic neutrophils after staining with **Apo-15** (100 nM) or FITC-Annexin V (25 nM) and image acquisition under the same experimental conditions (excitation/emission filters: 488 nm/525 nm). Data presented as mean values±SEM (n=3-4 independent cells from 3 independent experiments). Source data are provided as a Source Data file.

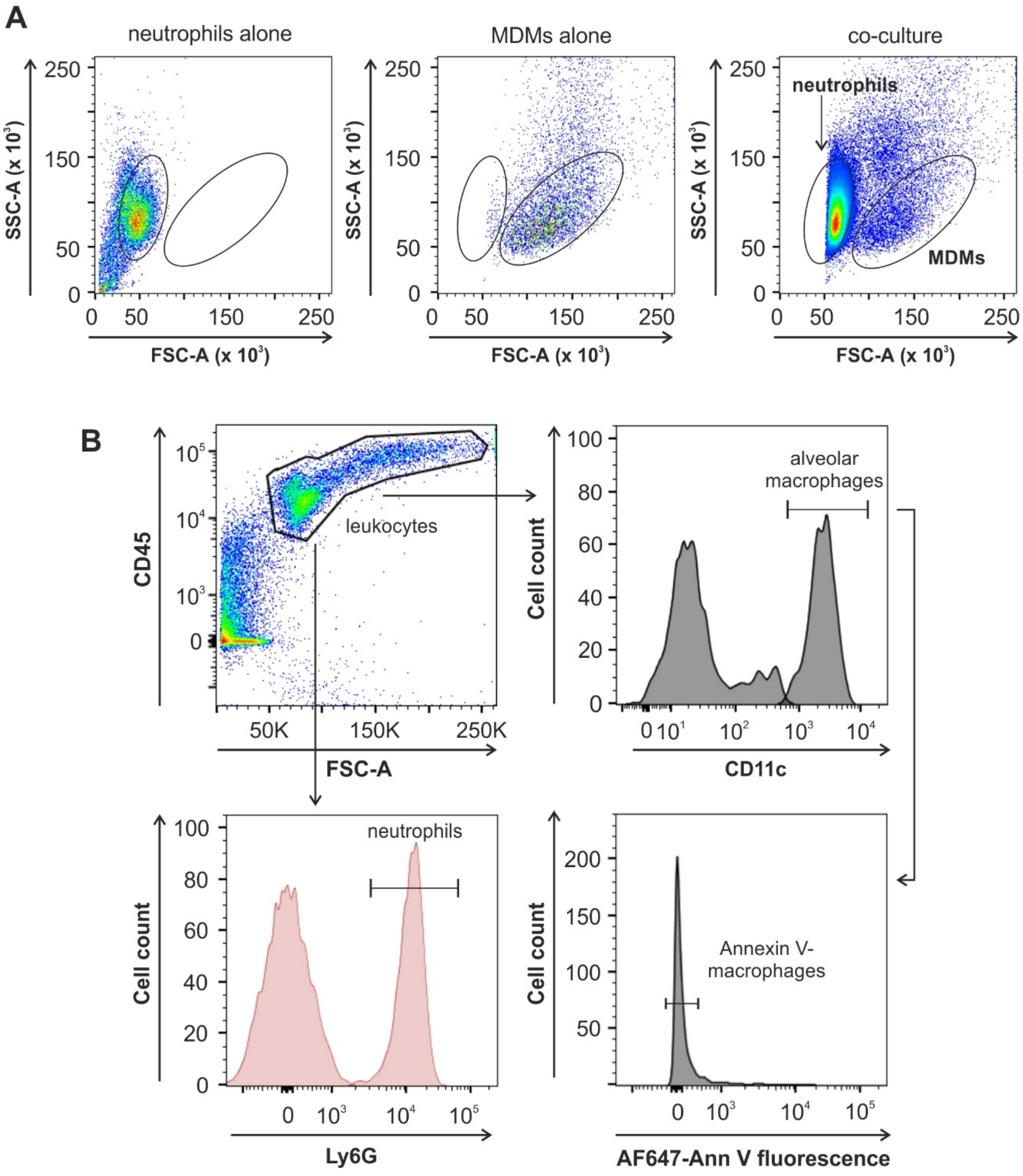

Supplementary Figure 15. A) Gating strategy for efferocytosis experiments in co-cultures of human neutrophils and monocyte-derived macrophages (MDMs). B) Gating strategy for neutrophils and alveolar macrophages isolated from BALF of mice that received LPS (20 μg mL^{-1}) and CDKi (AT7519, 30 mg kg^{-1}). Neutrophils were selected according to $CD45^{+}Ly6G^{+}CD11c^{-}$ expression, and viable macrophages were selected according to $CD45^{+}CD11c^{+}$ expression and further exclusion of Annexin V^{+} cells.

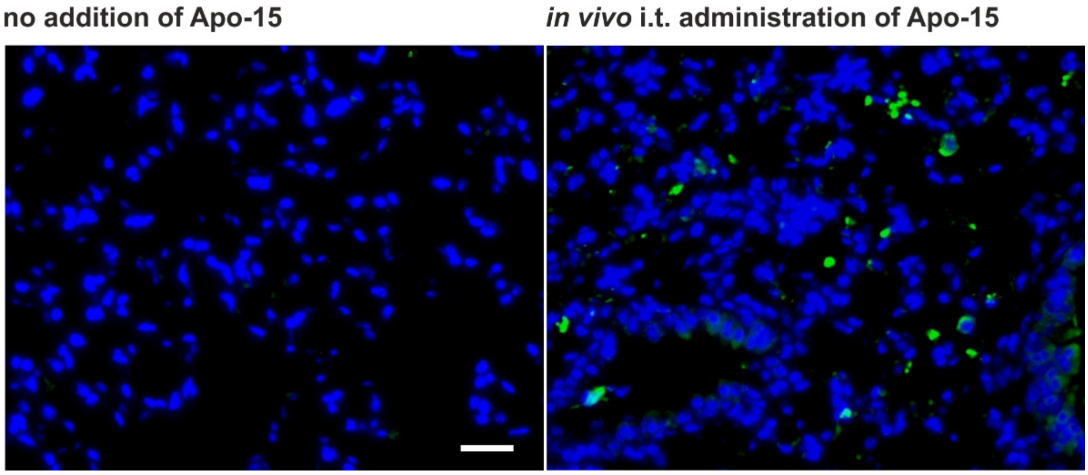

Supplementary Figure 16. Representative fluorescence microscope images of histological sections of lung tissue (5 μm thickness) from mice that had received LPS (20 μg mL^{-1}) and CDKi (AT7519, 30 mg kg^{-1}) without **Apo-15** (left) or with 5 μM **Apo-15** i.t. *in vivo* (right, green). Cells were co-stained with DAPI (18 μM, blue). Representative images from n=3 per group. Scale bar: 100 μm.

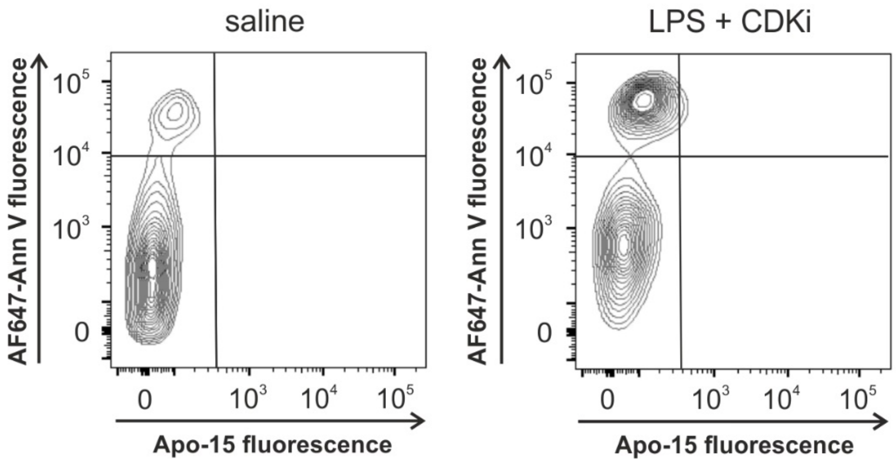

Supplementary Figure 17. Neutrophils were isolated from BALF of mice that received saline or LPS (20 μg mL^{-1}) plus CDKi (AT7519, 30 mg kg^{-1}) but not **Apo-15**. BALF cells were incubated with AF647-Annexin V *ex vivo* and data was acquired on 5L LSR flow cytometer. Representative histograms of neutrophils (gated as CD45$^+$Ly6G$^+$CD11c$^-$ as shown in Supplementary Figure 15B) show lack of fluorescence in the **Apo-15** channel even though apoptotic cells are clearly present as defined by Annexin V staining (n=4 independent experiments).

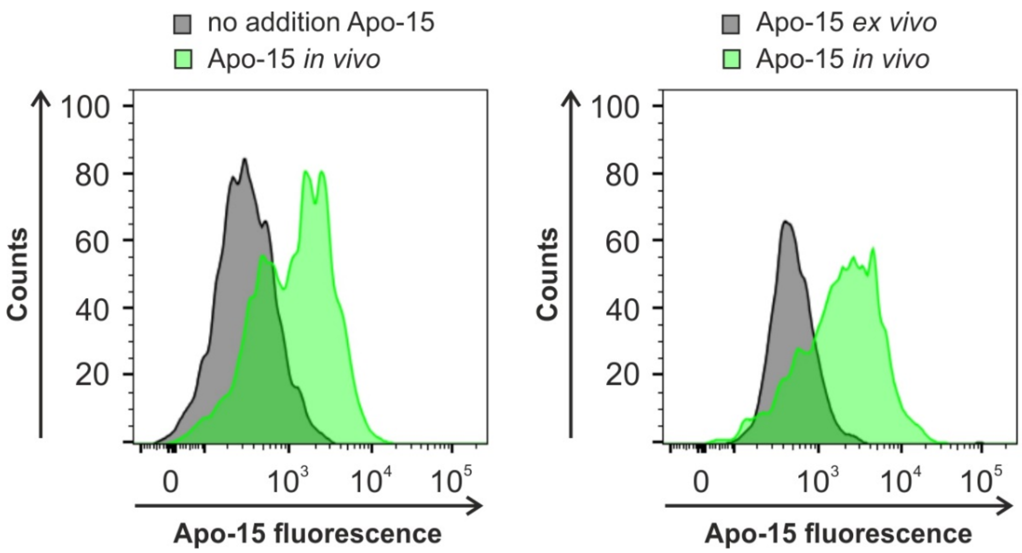

Supplementary Figure 18. Labeling of alveolar macrophages with **Apo-15** in vivo. Flow cytometric analysis of $CD45^+CD11c^+AnnexinV^-$ macrophages (gating strategy in Supplementary Figure 15B) from BALF of mice that received LPS (20 μg mL^{-1}) plus CDKi (AT7519, 30 mg kg^{-1}). Left) Representative histograms of macrophages that did not receive **Apo-15** (gray) or that received **Apo-15** (5 μM) (green) in vivo by intratracheal administration. Right) Representative histograms of macrophages that received **Apo-15** (5 μM) ex vivo (gray) or that received **Apo-15** (5 μM) in vivo (green) by intratracheal administration. Samples were analysed on a 5L LSR flow cytometer (n=4 independent experiments).

Supplementary Table 1. Characterization data of **Apo 0-15**.

**Compounds**	**$M_{calc.}$**	**$[M+H]^+_{exp.}$**	**tR (min)‡**	**Purity (%)‡**	**Yield (%)**
Apo-0	1324.5	1325.7	7.57	99	73
Apo-2	1321.7	1322.9	5.37	99	35
Apo-3	1282.7	1284.0	5.40	98	38
Apo-4	1243.7	1244.8	5.30	98	73
Apo-5	1321.7	1323.0	5.08	99	77
Apo-6	1321.7	1323.0	5.18	99	64
Apo-7	1321.7	1323.0	5.35	98	82
Apo-8	1321.7	1322.9	5.25	99	43
Apo-9	1175.7	1176.9	5.33	97	91
Apo-10	1161.9	1163.0	5.23	97	75
Apo-11	1379.8	1381.0	6.00	98	93
Apo-12	1437.7	1438.9	7.03	99	92
Apo-13	1263.9	1265.0	4.87	98	43
Apo-14	1205.9	1207.0	4.27	97	91
Apo-15	1310.7	1311.9	5.52	99	26

‡ Retention time and UV-based purity were determined under analytical HPLC conditions at 280 nm.

Supplementary Table 2. Binding kinetics for **Apo 0-15** (50 nM) as determined by flow cytometry, featuring the time required to reach 80% of maximum binding to apoptotic cells (BC_{80}).

**Compounds**	**R_1-R_5**	**Time BC_{80} (sec)**
Apo-0	EEEWW	n.a.
Apo-2	KKKWW	216
Apo-3	KKKWF	216
Apo-4	KKKFF	205
Apo-5	KWKWK	210
Apo-6	KKWWK	214
Apo-7	WWKKK	214
Apo-8	KWWKK	214
Apo-9	KKKLI	n.d.
Apo-10	KKKVI	n.d.
Apo-11	KKWWW	206
Apo-12	KWWWW	n.d.
Apo-13	KKKKW	n.d.
Apo-14	KKKKK	187
Apo-15	RKKWF	215

Supplementary Discussion

The evaluation of apopeptides shed light on some of the structural requirements for binding to apoptotic cell. In these assays, we observed a trend correlating overall peptide polarity or clog P with retention of staining (*Rt* in Fig. 1D). Markedly polar peptides (clog P < –4, **Apo-13** and **Apo-14**) bound to apoptotic neutrophils with fast kinetics but showed very low retention values. On the other hand, less polar peptides (clog P > –1, **Apo-11** and **Apo-12**) showed comparably stronger binding with slower rates. Apopeptides exhibiting labeling of apoptotic neutrophils with high retention values displayed balanced polarity (clog P values between –1 and –4). Consistent with the molecular requirements for binding negatively-charged phospholipids, **Apo-0** showed no binding to apoptotic cells, likely due to electrostatic repulsion between negative charges and carboxylic acid groups. However, the apopeptide library also highlighted the importance of interactions other than electrostatic ones. Apopeptides lacking hydrophobic aromatic residues (**Apo-9**, **Apo-10** and **Apo-14**) showed poor retention of staining after washing. Among aromatic residues, tryptophan (W) was preferred over phenylalanine (F) (**Apo-2** and **Apo-4**). In addition, the enhanced performance of **Apo-15** over **Apo-3** highlighted the importance of arginine (R) within the hydrophilic region. The guanidino group of arginine (R) results in increased polar surface area when compared to lysine (K), and therefore tighter electrostatic binding interactions. Finally, **Apo-8** appeared as the peptide with highest retention of apoptotic cell staining after washing. In that regard, the alternate hydrophobic/hydrophilic pattern might be important for binding to cell membranes. **Apo-8** was also the peptide with the highest background signal in viable cells.

Supplementary Methods

Materials and methods (Chemistry)

Coupling reagents COMU and Oxyma Pure were kindly provided from Luxembourg Biotechnologies. Fmoc-amino acids and 2-chlorotrityl chloride polystyrene resin were obtained from Iris Biotech and Merck Novabiochem respectively. Completion of peptide coupling was checked using a commercial Ninhydrin kit (AnaSpec) at 120ºC in a heating block inside a fume hood. Analytical HPLC system was performed on a Waters Alliance 2695 coupled to a Micromass ZQ (Masslynx 4.1) mass spectrometer and photodiode array detector using a Kinetex 150 × 4.6 mm^2 (5 μm) C_{18} column. Peptide purification was performed of a semi-preparative HPLC using UV detection and a Kinetex Axia 150 × 21.2 mm^2 (5 μm) C_{18} column. Eluents: H_2O (0.1% HCOOH) and ACN (0.1% HCOOH). NMR spectra were recorded on a 500 MHz spectrometer. Chemical shifts (δ) are reported in ppm. Multiplicities are referred by the following abbreviations: s = singlet, d = doublet, t = triplet, dd = double doublet, ddd = double double doublet, dt = double triplet, q= quartet and m = multiplet. HRMS (ESI positive) were obtained with a LTQ-FT Ultra (Thermo Scientific) mass spectrometer. clog P values were calculated using the Molinspiration desktop property calculator.

Chemical synthesis of apopeptides

Apopeptides were synthesized at 30 μmol scale starting from 2-chlorotrityl chloride polystyrene resin (loading:1.6 mmol g^{-1}). Fmoc-Gly-OH was introduced as first amino acid (1.4 eq.), by mixing the amino acid solution in DCM with DIPEA (3 eq.) for 10 min, then adding extra DIPEA (7 eq.) and stirring at r.t. for 55 min. Capping was performed by treatment with MeOH:DIPEA:DCM (10:5:85, ×3). Resins were washed with DCM (×5), DMF (×5) and Fmoc groups were removed with piperidine: DMF (2:8, 2×5 min),

collecting the filtrates for UV quantification of the piperidine-dibenzofulvene adduct at 301 nm. Subsequent couplings were performed using the corresponding amino acids (4 eq.), COMU (3.6 eq.), Oxyma (4 eq.), and DIPEA (8 eq.) with shaking for 90 min, with the exception of Fmoc-Trp(BODIPY)-OH, where 1.15 eq. were used, in conjuction with COMU (1.15 eq.), Oxyma (1.2 eq.) and DIPEA (3.0 eq) and coupling times of 2 h. Once peptide chain was elongated, cleavage from the resin was conducted by treatment with TFA:DCM (1:99, 5×1 min), followed by DCM washes (×5). Peptide filtrates were collected over a flask containing DCM (50 mL), prior to evaporation at reduced pressure, followed by reconstitution with H_2O:ACN (1:2) and lyophylization. Peptides were cyclized immediately thereafter without purification.

Head-to-tail cyclization of apopeptides was performed in DMF (55 mM) in presence of COMU (1.1 eq.) and DIPEA (2.5 eq.). Reactions were stirred in the dark for 2 h. Crudes were purified by semi-preparative HPLC with detection at 500 nm (flow: 10 mL min^{-1}) and the cyclic peptides were lyophilized. Side-chain protecting groups were removed by hydrogenation. Briefly, after performing 3 vacuum and N_2 purging cycles, H_2 (1.5 bar) was added to the system and the reactions were monitored by analytical HPLC. After 1-4 h, the solvent was removed, and the crudes were dissolved in MeOH prior to semi-preparative HPLC purification and lyophilization. Final apopeptides were obtained as orange solids in purities ≥ 97% (Supplementary Table 1).

Characterization data for Apo-15

^{1}H NMR (500 MHz,CD_3OD): δ 8.56 (s, 3H), 7.92 – 7.85 (m, 1H), 7.73 (t, *J* = 7.7 Hz, 1H), 7.68 (t, *J* = 1.7 Hz, 1H), 7.59 (d, *J* = 7.9 Hz, 1H), 7.55 (d, *J* = 7.9 Hz, 1H), 7.41 – 7.36 (m, 3H), 7.29 – 7.23 (m, 2H), 7.23 – 7.18 (m, 3H), 7.17 – 7.11 (m, 2H), 7.09 – 7.03 (m, 2H), 7.00 (s, 1H), 6.11 (s, 1H), 6.08 (s, 1H), 4.62 (m, 2H), 4.53 – 4.44 (m, 1H), 4.28 (dd, *J* = 9.0, 6.1 Hz, 1H), 4.21 (dd, *J* = 10.5, 4.9 Hz, 1H), 4.06 (dd, *J* = 9.2, 5.2 Hz, 1H), 3.97 (t, *J* = 6.9 Hz, 1H), 3.88 (d, *J* = 16.9 Hz, 1H), 3.42 (m, 2H), 3.25 – 3.11 (m, 3H), 3.10 – 3.02 (m, 1H), 2.92 – 2.82 (m, 3H), 2.73 – 2.62 (m, 1H), 2.51 (m, 8H), 2.12 – 1.78 (m, 3H), 1.68 (m, 4H), 1.55 (s, 3H), 1.54 (s, 3H), 1.50 – 1.26 (m, 7H), 1.02 (m, 2H) ppm.

^{13}C NMR (101 MHz, CD_3OD): δ 174.5, 174.3, 174.2, 173.9, 173.2, 171.8, 170.5, 158.7, 156.9, 144.7, 142.9, 138.2, 138.1, 137.8, 136.9, 136.1, 135.8, 132.8, 131.1, 130.7, 130.4, 130.1, 129.5, 128.6, 128.5, 127.9, 124.4, 123.3, 122.7, 122.5, 122.4, 120.4, 120.0, 119.9, 119.5, 112.5, 112.3, 111.5, 108.5, 58.8, 57.7, 56.2, 55.8, 55.3, 53.3, 43.9, 41.4, 40.5, 40.3, 39.7, 32.6, 31.3, 30.8, 30.0, 28.4, 27.5, 27.4, 26.4, 24.2, 23.4, 14.9, 14.6 ppm.

HRMS (ESI+) (m/z): $[M+Na]^+$ calcd. for $C_{70}H_{86}BF_2N_{16}O_7Na$, 1333.6741; found, 1333.6740.

A

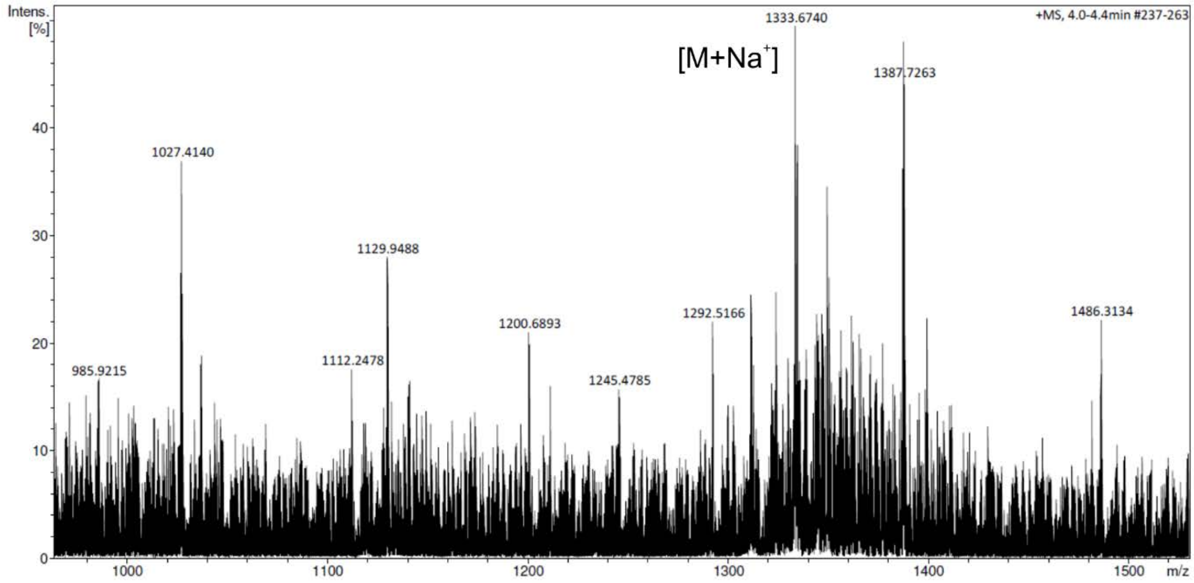
Intens. [%]
+MS, 4.0-4.4min #237-263
[M+Na+]
1333.6740
1387.7263
1027.4140
1129.9488
1200.6893
1292.5166
1486.3134
1112.2478
985.9215
1245.4785
m/z

B

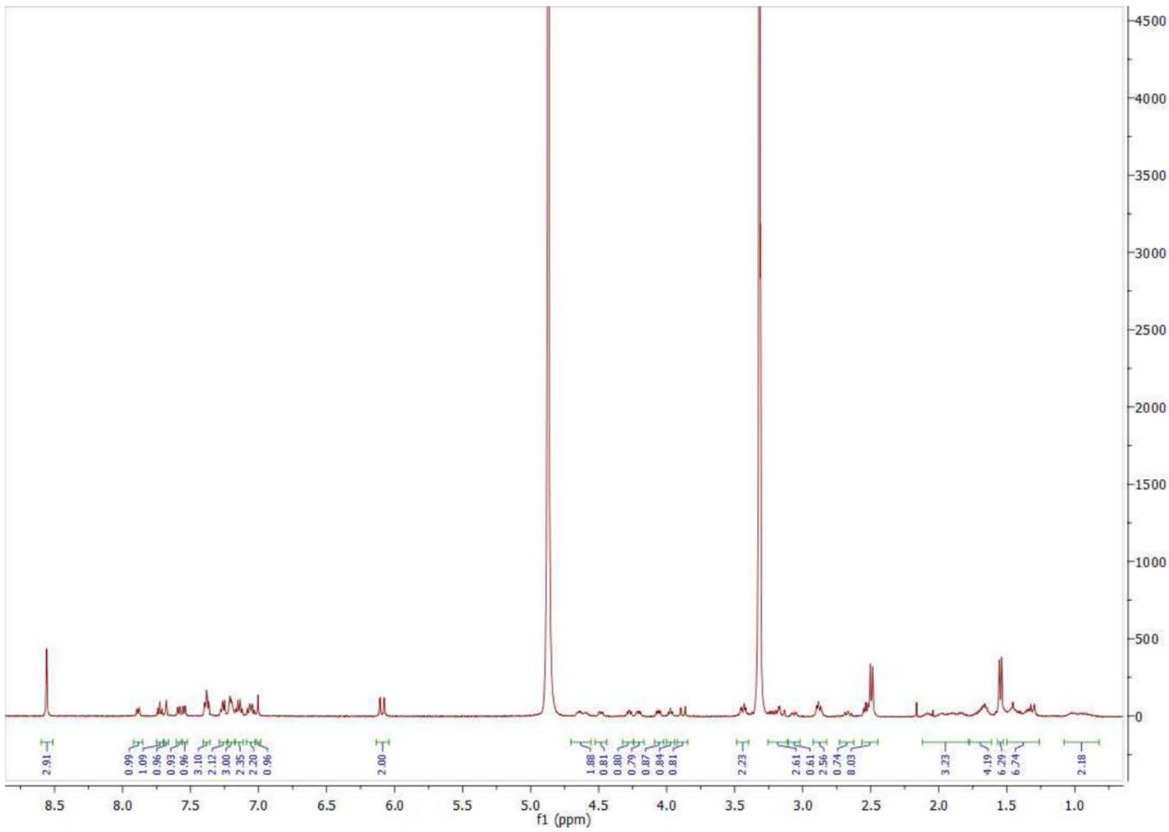
f1 (ppm)

C

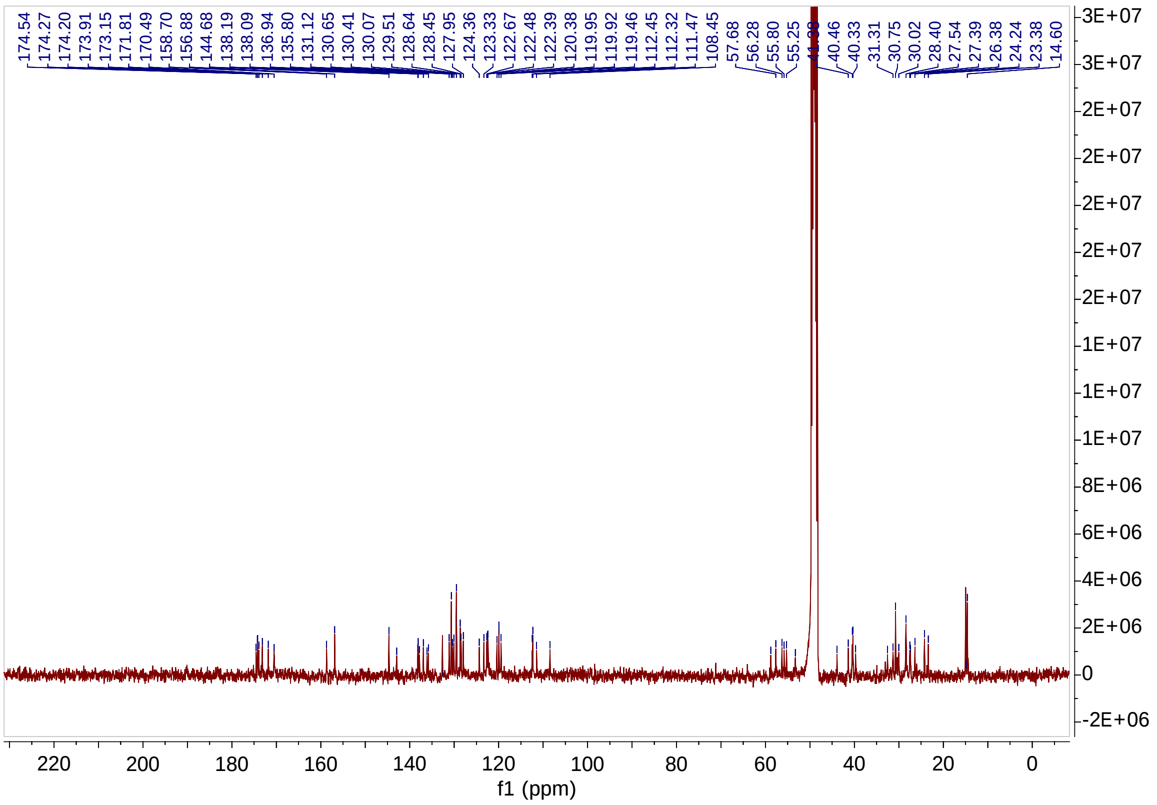

Chemical characterization data for **Apo-15**: A) High-resolution mass spectrum B) ^{1}H-NMR spectrum (500 MHz) in methanol-d4. C) ^{13}C-NMR spectrum (100 MHz) in methanol-d4.

Chemical stability of Apo-15

For protease stability assays, a stock of 5 µg mL^{-1} protease XIV (from *S. Griseus*) in PBS-citrate buffer at pH 7.1 was prepared. H_2O_2 was diluted to 100 µM in H_2O from a commercially available solution. **Apo-15** (50 µM) was incubated under both protease-rich and oxidative conditions for 24 h at 37 ºC in the dark and its chemical stability was determined at 0 h and 24 h by analytical HPLC under UV detection (Supplementary Figures 3 and 4).
