# Supplementary material for: A fluorogenic cyclic peptide for imaging and quantification of drug-induced apoptosis
Source: Nat Commun. 2020 Aug 12;11:4027. doi: 10.1038/s41467-020-17772-7 (PMC7423924; doi:10.1038/s41467-020-17772-7)
Supplement: Supplementary file 3 — Description of Additional Supplementary Files [file 41467_2020_17772_MOESM3_ESM.docx]

**Description of Additional Supplementary Files**

**File Name:** Supplementary Movie 1.

**Description:** Early-stage imaging of BL-2 cells undergoing apoptosis (Apo-15 channel). Human BL-2 cells were UV-irradiated (300 mJ cm^-2^) and incubated for 3 h at 37°C and 5% CO_2_. Time-lapse wash-free imaging was performed immediately after addition of **Apo-15** (100 nM) using a spinning-disk microscope (Andor) under live-cell conditions (λ_exc._: 488 nm, λ_em._: 525 nm). Images were acquired every 30 s for up to 15 min. Scale bar 10 µm.

**File Name:** Supplementary Movie 2.

**Description:** Early-stage imaging of BL-2 cells undergoing apoptosis (AF647-Annexin V channel). Human BL-2 cells were UV-irradiated (300 mJ cm^-2^) and incubated for 3 h at 37°C and 5% CO_2_. Time-lapse wash-free imaging was performed immediately after addition of AF647-Annexin V (5 nM) using a spinning-disk microscope (Andor) under live-cell conditions (λ_exc._: 633 nm, λ_em._: 670 nm). Images were acquired every 30 s for up to 15 min. Scale bar 10 µm.

**File Name:** Supplementary Movie 3.

**Description: Apo-15** labels subcellular material from apoptotic neutrophils. Neutrophils were isolated from the peripheral blood of healthy volunteers with Percoll density gradient and treated with *R*-roscovitine (20 µM) prior to imaging. Time-lapse wash-free imaging was performed immediately after addition of **Apo-15** (100 nM) using a spinning-disk microscope (Andor) under live-cell conditions (λ_exc._: 488 nm, λ_em._: 525 nm). Images were acquired every 1 s for up to 10 min. Scale bar: 10 µm.
