# Supplementary material for: A fluorogenic cyclic peptide for imaging and quantification of drug-induced apoptosis
Source: Nat Commun. 2020 Aug 12;11:4027. doi: 10.1038/s41467-020-17772-7 (PMC7423924; doi:10.1038/s41467-020-17772-7)
Supplement: Supplementary file 7 — Reporting Summary [file 41467_2020_17772_MOESM7_ESM.pdf]

nature research

Corresponding author(s): Marc Vendrell

Last updated by author(s): Jun 6, 2020

Reporting Summary

Nature Research wishes to improve the reproducibility of the work that we publish. This form provides structure for consistency and transparency in reporting. For further information on Nature Research policies, see Authors & Referees and the Editorial Policy Checklist .

Statistics

For all statistical analyses, confirm that the following items are present in the figure legend, table legend, main text, or Methods section.

n/a	Confirmed	
☐	☒	The exact sample size (n) for each experimental group/condition, given as a discrete number and unit of measurement
☐	☒	A statement on whether measurements were taken from distinct samples or whether the same sample was measured repeatedly
☐	☒	The statistical test(s) used AND whether they are one- or two-sided<br>*Only common tests should be described solely by name; describe more complex techniques in the Methods section.*
☒	☐	A description of all covariates tested
☒	☐	A description of any assumptions or corrections, such as tests of normality and adjustment for multiple comparisons
☐	☒	A full description of the statistical parameters including central tendency (e.g. means) or other basic estimates (e.g. regression coefficient) AND variation (e.g. standard deviation) or associated estimates of uncertainty (e.g. confidence intervals)
☐	☒	For null hypothesis testing, the test statistic (e.g. F, t, r) with confidence intervals, effect sizes, degrees of freedom and P value noted<br>*Give P values as exact values whenever suitable.*
☒	☐	For Bayesian analysis, information on the choice of priors and Markov chain Monte Carlo settings
☒	☐	For hierarchical and complex designs, identification of the appropriate level for tests and full reporting of outcomes
☒	☐	Estimates of effect sizes (e.g. Cohen's d, Pearson's r), indicating how they were calculated

Our web collection on statistics for biologists contains articles on many of the points above.

Software and code

Policy information about availability of computer code

Data collection	Standard commercial softwares were used for data collection.<br>Specifically, HPLC-MS: Masslynx 4.1; BD 5Laser LSR and BD 5Laser FACSAria: BD FACSDIVA V8.0; Leica SP8: Leica Application Suite X; Andor Spinning Disk: Andor IQ 3.6.1; FLIM: SymPhoTime; Intravital imaging: MicroManager 1.4; Synergy HT spectrophotometer: Gen 5.
Data analysis	Data analysis, including statistical analysis, was performed using Graphpad Prism 8.0. FlowJo V10 was used to analyze flow cytometry/FACS data collected using the 5L LSR as well as FACS Aria. Fluorescence image analysis was performed with Fiji ImageJ 1.52b. Intravital imaging data was analyzed with Imaris x64.

For manuscripts utilizing custom algorithms or software that are central to the research but not yet described in published literature, software must be made available to editors/reviewers. We strongly encourage code deposition in a community repository (e.g. GitHub). See the Nature Research guidelines for submitting code & software for further information.

Data

Policy information about availability of data

All manuscripts must include a data availability statement. This statement should provide the following information, where applicable:
- Accession codes, unique identifiers, or web links for publicly available datasets
- A list of figures that have associated raw data
- A description of any restrictions on data availability

The source data underlying Figs 1B, 2D, 3B, 3C, 3F, 4A, 4C, 5A, 5C and 5D and Supplementary Figs 1, 4, 10, 11, 12 and 14 are provided as a Source Data file. Additional data that support findings of this study are available from the corresponding authors upon reasonable request.

Field-specific reporting

Please select the one below that is the best fit for your research. If you are not sure, read the appropriate sections before making your selection.

[x] Life sciences [ ] Behavioural & social sciences [ ] Ecological, evolutionary & environmental sciences

For a reference copy of the document with all sections, see nature.com/documents/nr-reporting-summary-flat.pdf

Life sciences study design

All studies must disclose on these points even when the disclosure is negative.

Sample size	Sample/replicate size: The minimum number of experimental repeats was n=3, in line with current standards in the field. For all other experiments besides animal work, numbers for original data of the manuscript were based on previous experience. Sample size was not predetermined using statistical methods. For experiments in mice, sample size was calculated based on published experimental findings. Specifically, based on our previous work (Lucas et al., Mucosal Immunology 7 857-868 (2014)) in which CDKi treatment increased the number of apoptotic cells present in BALF from 0.66 +/-0.07x10E^4 to 4.1 +/-0.7 x10E^4, we calculated that using an unpaired t test with a two-sided alternative hypothesis and a critical p-value of 0.5, we would need 4 mice to be able to confirm a difference in the numbers of apoptotic cells between LPS only and LPS + CDKi treatment with over 90% power.
Data exclusions	No data was excluded.
Replication	For key findings, at least 3 independent experiments were performed in order to check the reproducibility of results. All attempts of replication were successful and included into statistical analysis. In some cases, e.g. induction of apoptosis in PLB985 cells, multiple assays, including caspase activation and cellular morphology, were used to validate findings. Data distribution was assumed to be normal, but this was not formally tested. Comparisons between groups were planned before statistical testing, target effect size was not predetermined by statistical methods. For samples taken from animals, typically 4 animals per treatment group as justified in our sample size summary. For representative images or flow cytometry plots, each experiment was successfully repeated at least three times under similar conditions.
Randomization	Animals were randomly assigned to experimental groups. No randomization method was used for remaining experiments. Cells from human peripheral blood have been allocated a specific donor coding system to protect donor anonymity and allow exact recording of experimental use without biases.
Blinding	Researchers were blinded for the quantification of Apo-15-positive events in lung slices using ImageJ. In flow cytometry experiments, the interpretation of all collected data was cross-checked with researchers blinded to the different treatment groups. For all other experiments using cell lines and human samples, data collection and analysis was not performed blinded. This was not needed given to treatment to induce cell death leads to easily phenotypically easy to distinguish viable and apoptotic cells.

Reporting for specific materials, systems and methods

We require information from authors about some types of materials, experimental systems and methods used in many studies. Here, indicate whether each material, system or method listed is relevant to your study. If you are not sure if a list item applies to your research, read the appropriate section before selecting a response.

Materials & experimental systems

n/a	Involved in the study	
[ ]	[x]	Antibodies
[ ]	[x]	Eukaryotic cell lines
[x]	[ ]	Palaeontology
[ ]	[x]	Animals and other organisms
[ ]	[x]	Human research participants
[x]	[ ]	Clinical data

Methods

n/a	Involved in the study	
[x]	[ ]	ChIP-seq
[ ]	[x]	Flow cytometry
[x]	[ ]	MRI-based neuroimaging

Antibodies

Antibodies used	PE-conjugated IgG2b(kappa) rat anti-mouse CD45 (Biolegend, cat#103106, Clone: 30-F11, Lot: B174317). This antibody reacts with all isoforms and both CD45.1 and CD45.2 alloantigens of CD45.<br>PerCP/Cy5.5-conjugated IgG2a(kappa) rat anti-mouse Ly6G (Biolegend, cat# 127616, Clone: 1A8, Lot: B181143). This antibody recognizes only Ly-6G, not the related Ly-6C molecule.<br>Pacific Blue-conjugated Hamster IgG anti-mouse CD11c (Biolegend, cat# 117322, Clone: N418, Lot: B213516).<br>Cleaved Caspase 3 (Asp175) (Cell Signaling, cat #9661S, Lot:43)<br>F(ab')2-Goat anti-Rabbit IgG (H+L) Cross-Adsorbed Secondary Antibody, Alexa Fluor 647 (Invitrogen, cat# A21246)<br>Furthermore, relevant isotype antibodies (rat IgG2b-PE; rat IgG2a-PerCP/Cy5.5 and Hamster IgG-Pac Blue) were used as controls

Validation

Validation was performed as indicated in the manufacturer's website. Antibodies have been rigorously tested and validated using IHC, Western blotting and Flow cytometry. The PE-conjugated rat anti-mouse CD45 has been used in 59 publications, please see: https://www.biolegend.com/en-us/products/pe-anti-mouse-cd45-antibody-100. PerCP/Cy5.5-conjugated rat anti-mouse Ly6G has been used in 40 publications, please see: https://www.biolegend.com/en-us/search-results/percp-anti-mouse-ly-6g-antibody-13351. Pacific Blue anti-mouse CD11c has been used in 26 publications, please see: https://www.biolegend.com/en-us/products/pacific-blue-anti-mouse-cd11c-antibody-3864. Anti-Cleaved Caspase 3 has been used in over 4600 publications, please see: https://www.cellsignal.co.uk/products/primary-antibodies/cleaved-caspase-3-asp175-antibody/9661. Moreover, the antibodies have been utilized in recent publications from our group (Felton et al., Journal Allergy Clinical Immunology 142, 1884-1893 (2018), Felton et al., Thorax, doi: 10.1136/thoraxjnl-2019-213204 (2020)).

Eukaryotic cell lines

Policy information about cell lines

Cell line source(s)

Primary Small Airway Epithelial Cells were obtained from ATCC.
BL-2 cells were provided by Prof. Chris Gregory (University of Edinburgh). This cell line was derived from a sporadic, Epstein-Barr virus-negative case of BL (G.M. Lenoir, M. Vuillaume, and C. Bonnardel, The use of lymphomatous and lymphoblastoid cell lines in the study of Burkitt's lymphoma. IARC scientific publications (1985), 309-318).
PLB985 cells were provided by Dr. Sonja Vermeren (University of Edinburgh). Cells were acquired from Lonza.

Authentication

Flow cytometry (CD19, MHC-Class II) was performed to confirm B-cell origin of BL-2 cells. PLB985 cells were confirmed as lacking the capacity to expose phosphatidylserine during apoptosis by lack of staining of Annexin V.

Mycoplasma contamination

Mycoplasma was not detected by staining for extranuclear DNA with Hoechst 33342.

Commonly misidentified lines
(See ICLAC register)

PLB985 cells are registered as a commonly misidentified cell line, where the contaminating cell line is the promyelocytic cell line HL-60. We confirmed that HL-60 cells were not cultured at the same time as maintaining PLB985 cells by lack of Annexin V staining in our PS-exposure experiments. HL-60 cells are reported not to expose PS following induction of apoptosis.
Primary Small Airway Epithelial Cells have not been registered as a commonly misidentified cell line. The use of PLB985 cells was essential as a cell line not exposing phosphatidylserine when undergoing apoptosis. An alternative would have been HL-60 cells, that is also a commonly misidentified cell line.

Animals and other organisms

Policy information about studies involving animals; ARRIVE guidelines recommended for reporting animal research

Laboratory animals

species: Mus musculus
strain: C57BL/6, MMTV-PyMT-C57BL/6
sex: female
age: 6-11 weeks (ALI model), 12-16 weeks (tumour model)
Housing: specific- pathogen- free facility with standard husbandry, Temperature 19-22 C, Humidity 45-55%, 12 h dark/light cycles

Wild animals

The study did not involve wild animals.

Field-collected samples

No field collected samples were used in the study.

Ethics oversight

UK Home Office license, Institutional Animal Care and Use Committee Cold Spring Harbour.

Note that full information on the approval of the study protocol must also be provided in the manuscript.

Human research participants

Policy information about studies involving human research participants

Population characteristics

Healthy male and female volunteers were used for this study (age range: 20-60 years). All donor samples were anonymized following blood sample collection. Information (sex, age, etc) was recorded but it was not made available to the researchers.

Recruitment

Healthy volunteers were randomly selected with availability (based on sample volume and frequency of donation) according to local ethical guidelines of the blood donor register. Information on donors was limited to age and gender and blinded for analysis of experiments. The selection of healthy participants was unbiased, with gender or age not impacting the extent of apoptosis nor the exposure of phosphatidylserine. Work complied with all relevant ethical regulations and informed consent was obtained. The study protocol was approved by the Accredited Medical Regional Ethics Committee (AMREC, reference number 15-HV-013) at the University of Edinburgh.

Ethics oversight

Work complied with all relevant ethical regulations and informed consent was obtained. The study protocol was approved by the Accredited Medical Regional Ethics Committee (AMREC, reference number 15-HV-013) at the University of Edinburgh.

Note that full information on the approval of the study protocol must also be provided in the manuscript.

Flow Cytometry

Plots

Confirm that:

- [x] The axis labels state the marker and fluorochrome used (e.g. CD4-FITC).
- [x] The axis scales are clearly visible. Include numbers along axes only for bottom left plot of group (a 'group' is an analysis of identical markers).
- [x] All plots are contour plots with outliers or pseudocolor plots.
- [x] A numerical value for number of cells or percentage (with statistics) is provided.

Methodology

Sample preparation	Sample preparation is described in detail in the Online Methods. Briefly, cells were washed once with HEPES-NaCl buffer prior to staining. Cells were then resuspended in HEPES-NaCl buffer with 2 mM CaCl2 or 2.5 mM EDTA containing peptides/antibodies/ stains and incubated (as detailed in the Figure legends) at 4 C. Antibody-stained samples were incubated with Fc-block prior to primary antibody addition (to reduce non-specific antibody binding) and then washed prior to analysis. As indicated when applicable, some experiments were performed where samples were analyzed without any washing.
Instrument	BD 5Laser LSR and BD 5Laser FACSAria
Software	Data collection was performed with FACSDIVA V8.0 software. Data was analyzed with FlowJo V10 software.
Cell population abundance	For flow cytometry experiments, 10,000 events were acquired for every population of interest.<br>For FACS experiments, Apo-15-positive and Apo-15-negative cells were sorted from samples containing approximately 5x10E6 cells.
Gating strategy	Firstly, singlet cells were gated in FSC-A and FSC-H plots to exclude debris and non-singlet events. Secondly, gates were constructed based on laser scatter properties (FSC-A and SSC-A). In BALF samples, additional gating was performed to identify subpopulations of cells (CD45+Ly6G+ for neutrophils and CD45+CD11c+ for macrophages). Macrophages were further gated on Annexin V negative cells. Gating is shown in the supplementary figures.

- [x] Tick this box to confirm that a figure exemplifying the gating strategy is provided in the Supplementary Information.
